# Location and content of counselling and acceptance of postpartum IUD in Sri Lanka

**DOI:** 10.1186/s12978-017-0304-7

**Published:** 2017-03-14

**Authors:** Mahesh Karra, David Canning, Sorcha Foster, Iqbal H. Shah, Hemantha Senanayake, U. D. P. Ratnasiri, Ramya Priyanwada Pathiraja

**Affiliations:** 1 Department of Global Health and Population, Harvard T.H. Chan School of Public Health, 665 Huntington Ave, Boston, MA 02115 USA; 2 Queens University Belfast, University Road Belfast, BT7 1NN Belfast, Northern Ireland UK; 3 Department of Obstetrics & Gynaecology, Faculty of Medicine, University of Colombo, Colombo, Sri Lanka; 4 Castle Street Hospital for Women, Colombo 08, Sri Lanka; 5 Faculty of Medical Sciences, University of Sri Jayewardenepura, Nugegoda, Sri Lanka

**Keywords:** Postpartum family planning, Postpartum IUD (PPIUD), Quality of care, Counselling, SRI Lanka

## Abstract

**Background:**

The immediate postpartum IUD (PPIUD) is a long-acting, reversible method of contraception that can be used safely and effectively following a birth. To appropriately facilitate the immediate postpartum insertion of IUDs, women must be informed of the method’s availability and must be counselled on its benefits and risks prior to entering the delivery room. We examine the relationship between the location and quality of antenatal counselling and women’s acceptance of immediate postpartum IUD (PPIUD) in four hospitals in Sri Lanka.

**Methods:**

Data were collected between January 2015 and May 2015. Modified Poisson regressions with robust standard errors are used to assess the relationships between place of counselling, indicators of counselling quality, and PPIUD uptake following delivery.

**Results:**

We find that women who were counselled in hospital antenatal clinics and admission wards were much more likely to have a PPIUD inserted than women who were counselled in field clinics or during home visits. Hospital-based counselling had higher quality indicators for providing information on PPIUD, and women were more likely to receive PPIUD information leaflets in hospital locations than in lower-tiered clinics or during home visits. Women who were counselled at hospital locations also reported a higher level of satisfaction with the counselling that they received. Receipt of hospital-based counselling was also linked to higher PPIUD uptake, in spite of the fact that women were more likely to be given information about the risks and alternatives to PPIUD in hospitals. The information about the risks of and alternatives to PPIUD, whether provided in hospital or in non-hospital settings, tended to lower the likelihood of acceptance to have a PPIUD insertion. Counselling in hospital admission wards was focused on women who had not been counselled at field clinics.

**Conclusions:**

The study findings call for efforts that improve the training of midwives who provide PPIUD counselling at field clinics and during the home visits. We also recommend that routine PPIUD counselling be conducted in hospitals, even if women have already been counselled elsewhere.

## Plain English summary

We examine the association between the location and quality of antenatal counselling and women’s acceptance of immediate postpartum IUD (PPIUD) in Sri Lanka. In particular, we assess the relationships between a woman’s place of counselling, the quality of counselling that she received, and her uptake of a PPIUD, which we measure by whether she had a PPIUD inserted following delivery.

Data on 13,731 women were collected in four hospitals between January 2015 and May 2015. We find that women who were counselled in hospital clinics and admission wards were much more likely to have a PPIUD inserted than women who were counselled in field clinics or during home visits. Hospital-based counselling was of higher quality for providing information on PPIUD, and women were more likely to receive PPIUD leaflets in hospitals. Women who were counselled at hospital locations also reported a higher level of satisfaction with the counselling that they received. Additionally, women who received counselling in hospital admission wards were more likely to not have been counselled at field clinics.

Receiving PPIUD counselling in hospitals was linked to higher uptake of PPIUD, even though women were more likely to be informed about the risks and alternatives to PPIUD in hospitals.

In conclusion: our results call for efforts that improve the training of midwives who provide PPIUD counselling in non-hospital settings. We also recommend that routine PPIUD counselling be conducted in hospitals, even if women have already been counselled elsewhere.

## Background

The World Health Organization (WHO) recommends that a woman wait at least 24 months after a live birth before attempting the next pregnancy in order to reduce the risk of adverse maternal, perinatal and infant outcomes [16, 22]. However, a woman’s fertility may return quickly following a birth, particularly if she is not exclusively breastfeeding, and some women may begin to ovulate within 4 to 6 weeks after delivery [9]. Given that most women are unaware of how soon fertility can return postpartum, they may not initiate contraception in a timely fashion and are likely to be at greater risk of unintended pregnancy. Findings from the Demographic and Health Surveys (DHS) show that in 17 of the 43 countries that conducted a DHS from 2005 to 2013, fewer than 10% of women reported using any method of contraception within 2 months of delivery [20]. Moreover, the provision of immediate postpartum family planning and of long-acting reversible and permanent methods to women who wanted no more children was low. Studies have also shown that many women who expressed a desire to postpone or limit childbearing do not receive any postpartum method because they fail to return for a postpartum visit, are lost to follow up, or face other barriers to receiving postnatal care [13, 19]. As a result, two out of three women are estimated to have an unmet need for contraception in the year following the birth of a child [15].

The provision of effective and immediate postpartum contraception, particularly long-acting reversible methods such as the copper Intrauterine Device (IUD), has been shown to reduce the risk of pregnancy and reveal higher continuation of use than other methods 6 months following a delivery [7]. The immediate postpartum IUD (PPIUD) is a long-acting, reversible method of contraception that can be used safely and effectively in the immediate postpartum period and even while the mother is breastfeeding [10]. The PPIUD offers a convenient and cost-effective contraceptive option to women who cannot return for follow-up visits because of distance, travel costs, and time constraints, or other barriers to access. Studies have shown that with adequate and effective provider training, expulsion and complication rates of PPIUD insertions are not dissimilar to those of interval IUDs, which are inserted 4 to 6 weeks after delivery [8]. However, the number of women in low-and middle-income settings who have accepted the PPIUD, as a proportion of women who delivered at facilities, has been low - a recent study found PPIUD acceptance among only 2.2% of immediate postpartum women in Ethiopia, 3.9% of immediate postpartum women in Guinea, 6.9% of immediate postpartum women in India, and 0.4% of immediate postpartum women in Pakistan, respectively [14]. To appropriately facilitate the immediate postpartum uptake of IUDs, women must be informed of the method’s availability and must be counselled on its benefits and risks, along with a similar information on other methods, prior to entering the delivery room and preferably during antenatal care [11].

The International Federation of Gynaecology and Obstetrics (FIGO), in collaboration with its nationally-affiliated Associations of Obstetricians and Gynaecologists, launched an initiative in 2014 to institutionalize PPIUD services as a routine part of antenatal counselling and delivery room services in six low- and middle-income countries: Sri Lanka, India, Kenya, Tanzania, Nepal and Bangladesh. The FIGO initiative was developed and launched to address the postpartum contraceptive needs of women by: 1) training community midwives, nurses, community health workers, and hospital staff (doctors and delivery unit staff) in the provision of counselling and postpartum family planning services; 2) institutionalizing the provision of counselling and postpartum family planning services, especially the PPIUD, as part of routine delivery services; and 3) ensuring continuity of PPIUD service provision, in which health providers who are trained in provision of PPIUD services are followed to determine whether they continue to provide these services even if they move to other facilities.

The main FIGO initiative in six countries was preceded by the implementation of the initiative in an initial set of six facilities in Sri Lanka, which FIGO rolled out with collaboration from its national affiliate, the Sri Lanka College of Obstetricians and Gynaecologists (SLCOG). This study, which evaluates the quality and effectiveness of the FIGO-SLCOG intervention’s counselling component on women’s receipt of PPIUD, is based on data gathered from four hospitals in Sri Lanka that were part of the first wave of facilities where the FIGO-SLCOG initiative was rolled out. In particular, we investigate the role of counselling on women’s acceptance of a PPIUD, and we measure a woman’s PPIUD acceptance by whether or not she had a PPIUD inserted after delivery.

The FIGO-SLCOG PPIUD intervention in Sri Lanka focuses on providing antenatal counselling to all women as well as with insertion of PPIUD immediately after delivery for women who consent during counselling and before delivery to have PPIUD. Previous studies in Peru [7] and Honduras [12] found that postpartum counselling, together with on-site provision of PPIUD, was effective in generating high uptake (by 25 and 17%, respectively) before discharge from hospital after delivery. More recently, a review of 35 interventions that aimed to improve uptake of postpartum family planning found that single antenatal counselling was largely ineffective, but intensive antenatal counselling and postnatal counselling, particularly in hospitals after delivery, have had a significant impact on uptake [3].

### The context

Since the end of its 26-year old civil conflict in 2009, Sri Lanka has made great economic progress and has begun the transition towards achieving middle-income country status [21]. Sri Lanka has a highly developed health system, particularly in the areas of obstetric and paediatric care. Antenatal care in Sri Lanka is free and comprehensive, and 99% of Sri Lankan women receive antenatal care at least once during pregnancy [23]. Antenatal care is provided at field clinics, at hospitals and hospital clinics, and through home visits by public health midwives (PHM). Low risk women who begin antenatal counselling at 6 to 8 weeks and who carry pregnancy to full term are encouraged to attend nine antenatal clinic visits, three home visits, and three antenatal classes over the course of their pregnancy [6]. Topics related to postpartum health and family planning are routinely discussed as part of these visits, particularly during the sixth and ninth clinic visits and the third antenatal class.

The total fertility rate in Sri Lanka fell slightly below the replacement level from 1995 to 2000, although it subsequently increased to 2.3 births per woman by 2006 [5]. Knowledge of contraceptives among Sri Lankan women of reproductive age is universal, and the median age of marriage and age of first birth, at 23.3 years and 25.1 years, respectively, are relatively late when compared to other low- and middle-income countries. The median birth interval in Sri Lanka is 52 months; however, 10.8% of birth intervals are less than 24 months and a further 16.1% are between 25 and 36 months. The provision of postpartum family planning may therefore serve to increase these short birth intervals and further improve maternal and child health.

Women in Sri Lanka report a high desire for limiting fertility. According to the 2006–7 Sri Lanka Demographic and Health Survey (DHS), 20.2% of Sri Lankan women with one child report wanting no more children, while the proportion rises to 75.0% for women with two children and 95.5% for women with three children [5]. Female sterilization is the most common method of family planning in Sri Lanka, with over 16% of married women of reproductive age reporting having been sterilized at the time of the survey. However, following concerns about the high prevalence of female sterilization and general controversy over forced female sterilization, legislation was passed in 1987 to restrict access to sterilization to women under 26 years unless they already had at least three living children and to women over 26 years unless: 1) they already had at least 2 living children; and 2) their youngest living child was over 2 years old. These restrictions implied that woman giving birth to their first or second child would not have access to sterilization immediately after delivery in Sri Lanka, which in turn has resulted in a shift in women’s use of family planning from permanent to temporary methods, and, in some cases, an increase in induced abortion [4]. The inclusion of the PPIUD in the method mix could be particularly appealing as a long acting method to: 1) women who would otherwise have chosen sterilization, but who are barred from doing so, and 2) women who prefer a long-acting reversible method free from user intervention, which is required for methods like the pill, condom, withdrawal, and abstinence.

The first wave of the FIGO-SLCOG PPIUD intervention in Sri Lanka provided training to health care providers in six large maternity and teaching hospitals in Sri Lanka. As part of the intervention, a PPIUD project facility coordinator was appointed in each hospital. In each of the six hospitals, senior professionals with experience in implementing new clinical practices were trained as “master trainers” in PPIUD counselling and insertion by FIGO and SLCOG. These master trainers were, in turn, tasked with the training of resident obstetricians and gynaecologists and the hospital’s delivery team in PPIUD counselling and insertion. The hospital facility coordinator was responsible for overseeing all PPIUD-related hospital activities and ensuring that PPIUD equipment was available in hospital delivery rooms. The training of doctors in each hospital included lectures, videos, web-based learning, master classes, and practical exercises. Doctors in each of the six hospitals received training on the correct insertion method of a PPIUD, which differs from traditional interval IUD placement in the uterine fundus, and were provided with Kelly forceps and complete PPIUD insertion kits. In order to successfully complete the training and be certified to insert PPIUDs, each doctor was required to make five correct insertions on the Mama-U model.

The training of midwives and nurses in counselling for PPIUD was undertaken as a means to ensure that the PPIUD counselling was integrated into existing family planning antenatal counselling services that are routinely provided at the hospital and field clinics and in home visits. Group training of nurses and midwives in each hospital district was arranged by SLCOG with support from local health administrators. PPIUD information leaflets were produced in both local languages, Sinhala and Tamil, and providers were asked to distribute PPIUD information leaflets to all women who were to be counselled on postpartum family planning during their antenatal visits.

To ensure continuity of care, Sri Lankan women are issued a pregnancy card upon their first antenatal visit. This pregnancy card records the number of antenatal visits for the pregnancy, any health concerns that the woman may be facing that could affect her pregnancy, any family planning counselling that the woman receives (including counselling on specific postpartum family planning methods), and any preferred family planning method that the woman is interested in receiving following her birth. A woman would then bring the pregnancy card to the hospital when she is admitted for her delivery.

Since the pregnancy card in use did not include PPIUD in the list of postpartum family planning methods, service providers who were trained as part of the intervention were instructed to place a white sticker on the pregnancy card that included PPIUD as an additional option to the standard set of postpartum methods. In addition, any woman who agreed to receive a PPIUD during antenatal counselling had a purple sticker affixed to her pregnancy card. Upon a woman’s arrival to the hospital for delivery, consent to receive a PPIUD was confirmed again prior to admission, even if the woman had received a purple sticker on her pregnancy card. In addition, hospital staff could also provide counselling on PPIUD prior to delivery to women who were admitted to the hospital, irrespective of whether or not they have been counselled previously. Upon confirmation of a woman’s eligibility to have a PPIUD insertion (e.g., that she did not exhibit any medical contraindications or experience a complication during delivery), a PPIUD was inserted by a doctor in the delivery room.

Counselling on PPIUD could take place in four locations, and women could report being counselled in one or more of these locations. In particular, women could be counselled during antenatal care visits at a primary health care field clinic or during home visits by a public health midwife. In addition, counselling on PPIUD could also be provided at hospital clinics to all women who chose to receive antenatal care at these clinics. Women can attend the hospital antenatal clinic by choice or may be referred to a hospital clinic if identified as a high risk pregnancy by a field clinic. Women could also receive counselling on PPIUD in the hospital ward after being admitted for delivery. In our analysis, a woman was coded as having received counselling in a location if she reported being counselled in that location, and her overall exposure to counselling in each of the four locations was captured using a set of four binary variables, which allowed us to assess how receipt of counselling by location is associated with acceptance of PPIUD.

After delivery, women in the six hospitals were interviewed about their experiences with the PPIUD counselling and their decision to take up the PPIUD. Questions on where they received PPIUD counselling and counselling quality, which included whether the woman was told about the benefits and risks of PPIUD, if they were informed of alternatives, if they were able to ask questions about PPIUD, if they were given the information leaflet on PPIUD, were administered. Women were also asked about their overall satisfaction with the PPIUD counselling that they had received. Finally, women were asked if they had a PPIUD inserted following their delivery. We assess where women were counselled, how the quality of counselling varied with location, and how the quality of counselling affected women’s uptake of PPIUD.

### Data and descriptive statistics

Data were collected for women who delivered in four of six hospitals^1^ in the first wave of the PPIUD intervention, namely: Castle Street Hospital for Women, De Soysa Maternity Hospital, Ragama Colombo North Teaching Hospital, and Galle Mahamodera Teaching Hospital between January 2015 and May 2015. Three of the four hospitals (Castle Street, De Soysa, and Ragama Colombo North) are located in Colombo, while Galle Mahamodera Teaching Hospital is located in Galle, about 119 km south of Colombo. Two data collection officers were assigned by each hospital to administer a questionnaire that collected information on each respondent’s sociodemographic background characteristics, the location and quality of antenatal counselling, and whether the respondent received a PPIUD following her delivery.

The goal was to interview all women who delivered in these four hospitals. All women who consented to receiving a PPIUD were interviewed in postnatal recovery wards by a team of data collection officers. Each team of data collection officers was comprised of medical officers and other hospital employees (nurses, senior midwives) who were trained in survey methods and interview techniques by the SLCOG project team. Given the volume of deliveries in the hospitals, some women who consented but did not receive a PPIUD were missed, particularly if they were discharged soon after delivery. A total of 16,398 women delivered in these hospitals and 14,049 women were interviewed over the 5 months in the four hospitals, of whom 1,015 consented to receiving a PPIUD following their delivery. We therefore have data on 85.7% of the total deliveries across the four hospitals. After adjusting for missing data on women who were interviewed, we are left with an analytic sample of 13,731 women.

Table 1 describes all of the variables that were used for the analysis. Table 2 presents the number of observations for each variable, the number of women who reported ‘yes’ for each binary variable, and the sample mean, standard deviation, minimum, and maximum values for each variable. The mean and standard deviation are calculated using inverse probability sampling weights to adjust for missing observations. These sampling weights are derived by calculating the ratio of women who were interviewed in a month in a hospital as a fraction of deliveries in the hospital that month. This procedure generates inverse probability weights on each observation that range from 1.00 to 1.74.

**Table 1:** Variable descriptions

Variable	Description
Age of mother	Woman’s age, in years.
Parity	The number of pregnancies that the woman has carried to term.
Number of living children	The number of living children before delivery
Woman counselled on PPIUD?	Whether the woman was counselled on PPIUD (1) or not (0).
Number of counselling locations	The number of locations where the woman was counselled on PPIUD
Counselling Location:	
Home	Whether the woman was counselled on PPIUD at home (1) or not (0).
Field Clinic (1 = yes)	Whether the woman was counselled on PPIUD at a field clinic (1) or not (0).
Hospital Clinic (1 = yes)	Whether the woman was counselled on PPIUD at a hospital clinic (1) or not (0).
Hospital Ward (1 = yes)	Whether the woman was counselled on PPIUD at a hospital ward (1) or not (0).
PPIUD Positive Counselling Quality Indicators:	
Could mention at least one risk?	Whether the woman could mention at least one risk from receiving a PPIUD (1) or not (0)
Client informed about alternatives?	Whether the woman was informed about alternatives to receiving PPIUD (1) or not (0)
Client given opportunity to ask questions?	Whether the woman was given an opportunity to ask questions about receiving a PPIUD (1) or not (0)
Given PPIUD information leaflet?	Whether the woman was provided the PPIUD information leaflet (1) or not (0)
PPIUD Negative Counselling Quality Indicators:	
Could she not mention at least one benefit?	Whether the woman could not mention at least one benefit from receiving a PPIUD (1) or not (0)
Dissatisfaction with PPIUD counselling?	Whether the woman was dissatisfied with the PPIUD counselling that she received (1) or not (0)
PPIUD Insertion:	
PPIUD inserted?	Whether a PPIUD was inserted (either intra-Caesarean section, post-placental, or within 48 h postpartum): 1 = yes, 0 = no

**Table 2:** Descriptive statistics, weighted means

VARIABLES	*N*	*N*_1_	Min.	Max.	Mean	Standard deviation
Age of mother	13693		14.0	48.0	28.682	5.509
Parity	13731		1.0	9.0	2.012	1.100
Number of living children	13731		0.0	12.0	1.606	0.966
Woman counselled on PPIUD?	13416	13362	0.0	1.0	0.996	0.060
Number of counselling locations	13731		0.0	4.0	1.871	1.173
Counselling Location:						
Home (1 = yes)	13416	4119	0.0	1.0	0.307	0.461
Field Clinic (1 = yes)	13160	9449	0.0	1.0	0.718	0.450
Hospital Clinic (1 = yes)	13160	5475	0.0	1.0	0.416	0.493
Hospital Ward (1 = yes)	13156	5683	0.0	1.0	0.432	0.495
PPIUD Positive Counselling Quality Indicators:						
Could mention at least one risk?	13117	11438	0.0	1.0	0.872	0.334
Client informed about alternatives?	13121	12281	0.0	1.0	0.936	0.245
Client given opportunity to ask questions?	13073	12001	0.0	1.0	0.918	0.275
Given PPIUD information leaflet?	13056	6450	0.0	1.0	0.494	0.500
PPIUD Negative Counselling Quality Indicators:						
Could not mention at least one benefit?	13117	1102	0.0	1.0	0.084	0.277
Dissatisfaction with PPIUD counselling?	12659	1342	0.0	1.0	0.106	0.307
PPIUD Insertion:						
PPIUD inserted?	13731	672	0.0	1.0	0.049	0.216
Total	13731					

*Notes*: *N*_1_ is the proportion of respondents who responded “yes” to the binary variable and is calculated by multiplying the sample size *N* by the weighted mean. Sample means are weighted by inverse probability weights

**Fig. 1:**
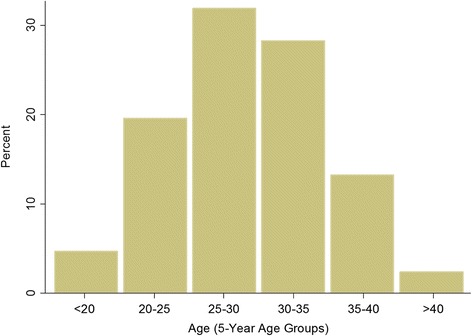
Percentage Distribution of Women by Age Groups, Weighted

**Fig. 2:**
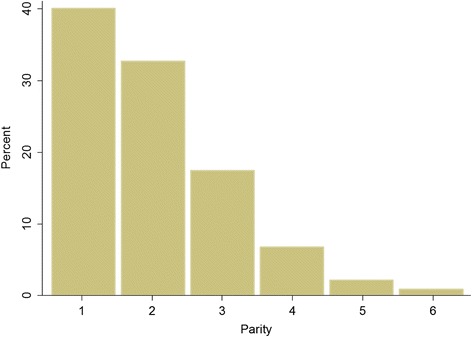
Percentage Distribution of Women by Parity, Weighted

The average age of women in the sample is 28.7 years and the average parity is 2.0 children. Figures 1 and 2 show the weighted distribution of women in the sample by age and by parity, respectively.

Almost all women (99.6%) were counselled at least once on PPIUD prior to delivery. On average, women report being counselled at 1.8 different locations. The most common place for counselling is the field clinic (for 71.8% of women), although receiving PPIUD counselling in the home, at the hospital antenatal clinic, and in the hospital ward, respectively, is also common.

The quality of antenatal counselling for PPIUD is assessed on the basis of respondent knowledge of the method. Respondents were asked to mention at least one specific benefit of the PPIUD and were recorded as knowing of a benefit if their answers corresponded to one of benefits of PPIUD that were listed in the training of providers. Similarly, women were asked to mention at least one risk of PPIUD and the response was evaluated against the list of risks providers had been trained to counsel on. In our sample, 87.2% of women were able to identify a risk of PPIUD, while 8.4% of women were unable to identify any benefits of PPIUD. Women were asked if there were informed about alternatives to PPIUD and if they were given the opportunity to ask questions about PPIUD during counselling. They were also asked if they had received a PPIUD leaflet and if they were satisfied with the PPIUD counselling they received. Respondent satisfaction with counselling is measured as a binary indicator in our analysis, in which reporting of counselling being “very good” or “good” is categorized as being satisfied with counselling, while counselling that is reported as “barely satisfactory,” “poor,” or “very poor” is categorized as not being satisfied. The reliability of this categorization method to accurately assess the quality of family planning counselling provided has been previously discussed in the literature [18]. Reported levels for all of these quality indicators were high except for receiving a PPIUD leaflet; only 49.4% of women reported receiving a PPIUD leaflet. Finally, 672 women (4.9%) had a PPIUD inserted following their delivery.

## Methods

We use modified Poisson regressions with robust standard errors to analyse our primary relationships of interest. This approach has the advantage that the reported regression coefficients can be interpreted as relative risks, whereas results from logistic regressions are presented as odds ratios, which are more difficult to interpret [24]. In order to correct for the sample selection, we assume that data collection officers collected data on all women who consented at any time to receive a PPIUD but only on a fraction of those who did not consent. We therefore take the sampling probability for each non-consenting woman who delivered in a given hospital as the ratio of the number of interviews to the number of total deliveries in that month in that hospital, and we weight all our regressions with these weights to adjust for the sample selection. All analyses were performed using STATA, version 13.

## Results

**Fig. 3:**
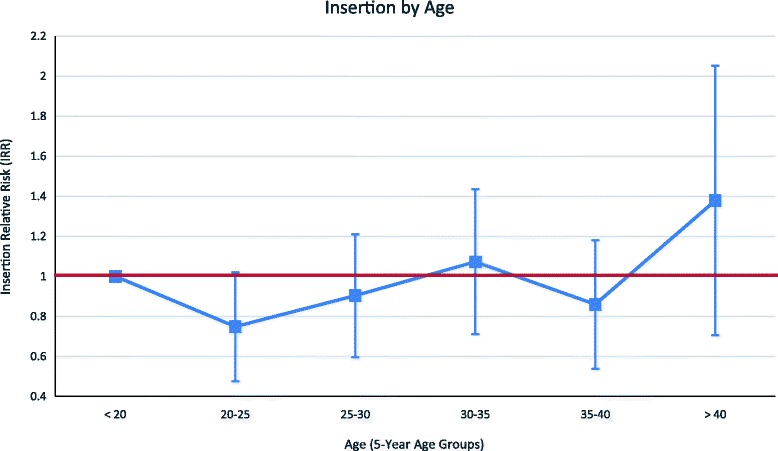
Relative Likelihood of PPIUD Insertion by Age, Weighted. Notes: Women who are younger than 20 are the reference category. The red line indicates the null relative risk value of 1

**Fig. 4:**
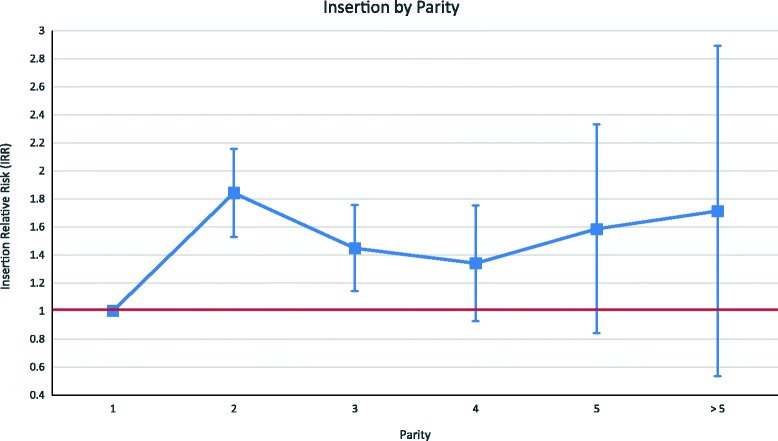
Relative Likelihood of PPIUD Insertion by Parity, Weighted. Notes: Women with parity 1 are the reference category. The red line indicates the null relative risk value of 1

Figures 3 and 4 plot the unadjusted relative risk and 95% confidence intervals of having a PPIUD insertion by age group and parity, respectively. In these analyses, women who were younger than 20 and women of parity 1 were taken as the reference groups. The results indicate that there is no significant relationship between age and acceptance of a PPIUD; on the other hand, women of parity 2 and 3 appear to be more likely to take up a PPIUD.

Table 3 provides regression results on the determinants of PPIUD uptake. Each column in the table presents relative risk estimates from a different regression specification, with insertion of a PPIUD being the binary outcome variable of interest. We report the effects of the location of counselling as well as the effects of counselling quality. All regressions control for hospital and month fixed effects as well as women’s age (by 5-year age group) and parity. All regressions also use women who received no counselling as the reference category. Column 1 of Table 3 reports the effect of counselling location on uptake. Women who received counselling in either hospital clinics or hospital wards were much more likely to have a PPIUD inserted (relative risks of consent exceed one) compared to women who were counselled at field clinics or at home by a PHM (relative risks less than one).

**Table 3:** Determinants of PPIUD insertion

VARIABLES	PPIUD insertion	PPIUD insertion	PPIUD insertion
PPIUD Positive Quality Indicators			
Could mention at least one risk?		0.133***	0.0473***
		(0.0526)	(0.0203)
Client informed about alternatives?		0.215***	0.674
		(0.0893)	(0.300)
Client given opportunity to ask questions?		1.321*	1.220
		(0.189)	(0.180)
Given PPIUD information leaflet?		8.367***	5.633***
		(1.064)	(0.768)
PPIUD Negative Quality Indicators			
Could not mention at least one benefit?		1.58e-07***	7.92e-08***
		(3.52e-08)	(2.36e-08)
Dissatisfaction with PPIUD counselling?		0.0242***	0.0485***
		(0.0245)	(0.0487)
Counselling Location			
Home	0.160***		0.184***
	(0.0271)		(0.0278)
Field Clinic	0.173***		0.152***
	(0.0231)		(0.0179)
Hospital Clinic	1.931***		0.975
	(0.200)		(0.0942)
Hospital Ward	4.372***		2.139***
	(0.441)		(0.219)
Observations	12971	12316	12295

*** *p* < 0.01, ** *p* < 0.05, * *p* < 0.1

*Notes*: The unit of observation is a woman. Incidence rate ratios are presented with robust standard errors in the parentheses below. Results are from a modified Poisson regression that includes age, parity, and hospital controls. Regressions are also weighted by the inverse of the proportion sampled in order to correct for the fact that enumerators failed to collect information for around 14.3% of the total number of deliveries across the four hospitals. Counselling received is whether counselling was received at this location type or not. Information on PPIUD is whether the respondent received the information on this element of counselling or not

Insertion rates for PPIUD are influenced by the quality of counselling, as shown in Column 2 of Table 3. Having the opportunity to ask questions during counselling increased the likelihood that a woman would take up a PPIUD. Moreover, women who received a PPIUD information leaflet were significantly more likely to have a PPIUD inserted following their delivery. On the other hand, not being able to identify any benefits of PPIUD, being able to identify a risk of PPIUD, or being informed about alternative methods lowers insertion rates. In addition, women who were dissatisfied with PPIUD counselling were 97.6% less likely to take up a PPIUD. In Column 3 of Table 3, we include both the location and quality of the counselling as explanatory variables, and we find that the results qualitatively hold when compared to those in columns 1 and 2. In particular, the associations between location type and PPIUD insertion rates remain significant. Where PPIUD counselling is administered appears to affect PPIUD insertion rates in ways other than simply through the quality of counselling as measured by our quality indicators. It may be that there are additional unmeasured quality measures we are not capturing.

In Table 4, we report the relationship between the location of counselling and our quality indicators. We find that there exists a consistent pattern between counselling location and quality, with counselling quality being higher when women are counselled in hospital clinics or in hospital wards rather than in field clinics or at home. In general, as shown in Table 2, the average of quality indicator scores is high, and the measured quality differentials across locations found in Table 3 are quite small. For example, women counselled at the hospital ward are 13% more likely to know of a risk to PPIUD, while women counselled at a field clinic are 22% less likely to know of a risk.

**Table 4:** Determinants of PPIUD Counselling Quality Indicators by location

Quality indicators	Leaflet	Risks	Alternatives	Questions	No benefits	Dissatisfaction
Counselling location:						
Home	0.868***	1.034	0.904***	0.923**	1.353	2.698***
	(0.0338)	(0.0279)	(0.0224)	(0.0288)	(0.402)	(0.603)
Field Clinic	0.946**	0.784***	0.973***	0.923***	0.454***	1.499***
	(0.0247)	(0.00781)	(0.00688)	(0.00718)	(0.139)	(0.178)
Hospital Clinic	2.535***	1.128***	1.003	1.068***	0.0405***	0.998
	(0.0665)	(0.0102)	(0.00654)	(0.00712)	(0.0152)	(0.104)
Hospital Ward	2.093***	1.130***	1.045***	1.052***	0.100***	0.382***
	(0.0427)	(0.00653)	(0.00475)	(0.00561)	(0.0327)	(0.0503)
Observations	12930	12963	12962	12929	12963	12371

*** *p* < 0.01, ** *p* < 0.05, * *p* < 0.1

*Notes*: The unit of observation is whether respondent was informed of binary indicators of quality: PPIUD benefits, risks, alternatives, opportunity to ask questions, receipt of PPIUD leaflet, and satisfaction. Incidence rate ratios are presented with robust standard errors in the parentheses below. Results are from a modified Poisson regression that includes age, parity, and hospital controls. Regressions are also weighted by the inverse of the proportion sampled in order to correct for the fact that enumerators failed to collect information for around 14.3% of the total number of deliveries across the four hospitals

One place where we see a very large effect of location is on whether a respondent received a PPIUD information leaflet at the time of counselling - we find that PPIUD information leaflets are given to only around half of respondents, and a woman was more than twice as likely to receive an information leaflet if she was counselled at the hospital. Importantly, we find that the higher likelihood of a woman to take up a PPIUD if she was counselled in a hospital clinic or ward is not because she was more likely to receive lower quality counselling (e.g., she was less likely to be informed about the risks associated with PPIUD or the alternatives to receiving a PPIUD) in hospital settings.

In Sri Lanka, women usually first receive antenatal care at field clinics or at home, and they later visit hospital clinics and wards (where they may receive additional counselling) at the time of delivery. Our earlier results have suggested that counselling in hospital settings is of higher quality and is more likely to increase PPIUD acceptance than counselling that is more likely to be received earlier and in non-hospital locations. In Table 5, we conduct regressions to determine who is counselled at the hospital clinical and wards. We find that women who were counselled at field clinics are around half as likely as others to be counselled in the hospital. In contrast, women who were not counselled at the field clinic are much more likely to be counselled in the hospital, and this accounts for the very high proportion of women who were counselled on PPIUD in at least one location.

**Table 5:** Determinants of hospital counselling

Location of counselling:	Hospital clinic	Hospital ward
Home	1.132***	1.120**
	(0.0519)	(0.0511)
Field Clinic	0.570***	0.491***
	(0.0156)	(0.0122)
Hospital Clinic		1.224***
		(0.0345)
Observations	12973	12971

*** *p* < 0.01, ** *p* < 0.05, * *p* < 0.1

*Notes*: The units of observation are a) whether a respondent was counselled at a hospital clinic and b) whether a respondent was counselled on a hospital ward. Incidence rate ratios are presented with robust standard errors in the parentheses below. Results are from a modified Poisson regression that includes age, parity and hospital controls. Regressions are also weighted by the inverse of the proportion sampled in order to correct for the fact that enumerators failed to collect information for around 14.3% of the total number of deliveries across the four hospitals

## Discussion

In this study, we assess the relationship between the location and quality of counselling on the acceptance and uptake of PPIUD among women who delivered in four large hospitals in Sri Lanka. When assessing the quality of counselling that women received over their pregnancies, we find that information on PPIUD that was provided as part of hospital-based counselling was of higher quality, and women were more likely to receive PPIUD information leaflets in hospital locations than as part of counselling in non-hospital settings. Women who were counselled at hospital locations also reported a higher level of satisfaction with the counselling that they received. The information about the risks of and alternatives to PPIUD, whether provided in hospital or in non-hospital settings, tended to lower the likelihood of acceptance to have a PPIUD insertion. Finally, we find that receipt of counselling in hospital admission wards was higher among women who had not been counselled at field clinics.

### Study limitations

Because of data constraints, we were unable to examine the association between the frequency of counselling a woman was exposed to over the course of her pregnancy and her likelihood of taking up a PPIUD. In addition, it was difficult to precisely assess the quality of counselling at field clinics when we did not have more structural and technical indicators of quality on these clinics. Moreover, it is possible that women who access antenatal care at different locations may differ in terms of socio-demographic background than women who access antenatal care at one location. Controlling for factors such as a woman’s education level, socioeconomic status, her previous family planning use, social network characteristics, and other factors would provide more robust estimation of the association between uptake of postpartum IUD by location of counselling. However, since the survey was administered to immediate postpartum women in postnatal wards, the data collection team was encouraged to keep the survey instrument as short as possible in order to minimize any inconvenience caused to women as they recovered from their recent deliveries. As a result, data on these socio-demographic variables and on other factors, including women’s preferences for family planning and their satisfaction with the PPIUD services that they received, were not collected and, therefore, we were unable to consider them in our analysis.

## Conclusions

Consistent with previous research findings, we observe that high quality counselling is important for the acceptance of postpartum family planning, particularly PPIUD [1, 2, 17]. In the Sri Lankan context, we demonstrate that high quality counselling, as assessed by specified indicators, was more likely to be provided in hospital wards and hospital clinics, and we find that this receipt of hospital-based counselling was also linked to higher PPIUD uptake, in spite of the fact that women were more likely to be given information about the risks and alternatives to PPIUD in hospitals.

In Sri Lanka, counselling for postpartum family planning is primarily provided at field clinics and during home visits. While quality indicators for PPIUD counselling in these settings was generally good, there is considerable room for improvement, perhaps through the re-training of field midwives and local medical staff, through the provision of additional training workshops to ensure that all midwives are adequately trained, and by increasing general awareness of PPIUD service availability. From a health systems perspective, it is also important to focus on strengthening the monitoring capacity of these lower-tiered clinics to be able to effectively follow up with women who have received PPIUD counselling and insertion services after delivery. In the long run, the PPIUD may be included in the standard information leaflet for women on postpartum family planning methods, but until such time as that happens, specific PPIUD information leaflets should be available and should be given to women as part of their counselling. Finally, we find that the level of counselling in Sri Lankan hospitals has shown to be of higher quality and has been more effective to date in generating awareness and acceptance of PPIUD, and we therefore recommend that hospital based counselling be universal and not reserved for only those women who were not counselled in other locations. This is in line with the finding of Cleland et al. [3], who conclude that counselling in hospitals increase the uptake of postpartum family planning.

## Footnotes

1 The other two hospitals collected data only on women who accepted PPIUD and not for all women who delivered.
